# Ophthalmic Treatment and Vision Care of a Patient with Rare Ring Chromosome 15: A Case Report

**DOI:** 10.1155/2014/285132

**Published:** 2014-06-03

**Authors:** Lidia Puchalska-Niedbał, Stanisław Zajączek, Elżbieta Petriczko, Urszula Kulik

**Affiliations:** ^1^Department of Ophthalmology, Pomeranian Medical University, Aleja Powstaców Wielkopolskich 72, 70-111 Szczecin, Poland; ^2^Cytogenetic Unit, Department of Pathology, Pomeranian Medical University, Aleja Powstaców Wielkopolskich 72, 70-111 Szczecin, Poland; ^3^Department of Paediatrics, Endocrinology, Diabetology, Metabolic Disorders and Cardiology, Pomeranian Medical University, Aleja Powstaców Wielkopolskich 72, 70-111 Szczecin, Poland

## Abstract

*The Aim*. Ring chromosome 15 is a very rare genetic abnormality with a wide spectrum of clinical findings. Up to date, about 50 cases have been documented, whereas no reports on ophthalmological treatment of such patients have been published. The aim of this study is not only to describe a new patient, but also, for the first time, to present the results of nonoperative management of divergent strabismus. * Material and Methods*. We present an amblyopic patient with 46,XX, r(15) karyotype: treated conservatively for exotropia of 60 prism diopters. The management consisted of refractive and prismatic correction, eye occlusion, and orthoptic exercises between the age of 15 months and 8 years. * Results*. The deviation angle of exotropia was decreased to 10 prism diopters, the visual acuity improved to 1.0 in both eyes (Snellen chart) and the fixation pattern was normal. The prisms enabled permanent symmetrical stimulation of the retina, which lead to a development of normal single binocular vision (Maddox test, filter test, and synoptophore tests). * Conclusions*. Parental karyotype was normal; the analysis of a three-generation pedigree has shown no genetic abnormalities or pregnancy losses so the child's karyotype anomaly was classified as * de novo that is* a single occurrence of this type of chromosomal disorder in this family. Strabismus in ring chromosome 15 patients is a difficult condition to manage, although success may be achieved.

## 1. Introduction

Ring chromosome 15 r(15) is a rare anomaly both in “pure” and in mosaic forms [1]; so far, only ~50 cases were described [2], and only three cases so far have been reported in prenatal diagnosis [2–4] and just one with a twenty-year cytogenetic and molecular followup [5]. Previous studies showed that ring chromosome 15 results in a varied and unspecific phenotype [6, 7]. However, a recurrent form has been characterized by growth deficiency, mental retardation, and characteristic dysmorphic features. Diagnosis has been problematic as similar clinical findings have also been noted in patients with other syndromes [2, 4, 8].

The purpose of this case study is to present for the first time the state of visual acuity and formation of normal binocular vision in the squinting eye with initial eccentric fixation and amblyopia by means of long-term conservative treatment [9]. As far as we know, there have been no previous reports published on ophthalmic treatment and vision care among ring chromosome 15 patients.

## 2. Case Study

A 15-month-old girl was referred to the pediatric outpatient department and the clinic of ophthalmology for treatment of divergent strabismus of the right eye. The girl underwent treatment between the age of 15 months and 8 years.

Her medical history revealed that she was born at term from a 5th uneventful pregnancy and delivery (10 points on the Apgar score), with a weight of 2900 g (10–25 percentile), length of 49 cm (50 percentile), and a normal head circumference of 33 cm (10 percentile). The girl is a daughter of healthy, unrelated parents. Her mother is 37 years old and 172 cm tall (+1.0 SD) and her father is 29 years old and 188 cm tall (+1.4 SD). The girl has four older healthy siblings; three sisters—17 years old with a height of 180 cm (+2.3 SD), 14 years old with a height of 175 cm (+2.2 SD), and 12 years old with a height of 164 cm (+1.7 SD). Her only brother is 7 years old with a height of 130 cm (+1.3 SD). During the neonate period she was diagnosed with atrial septum defect type II, which was corrected at the age of 12 months. At the age of 3 months the girl was also qualified for orthopedic treatment due to equinovarious feet. At 15 months, because of the dysmorphic features and growth retardation, she was diagnosed in the department of pediatric endocrinology and department of clinical genetics.

**Figure 1:**
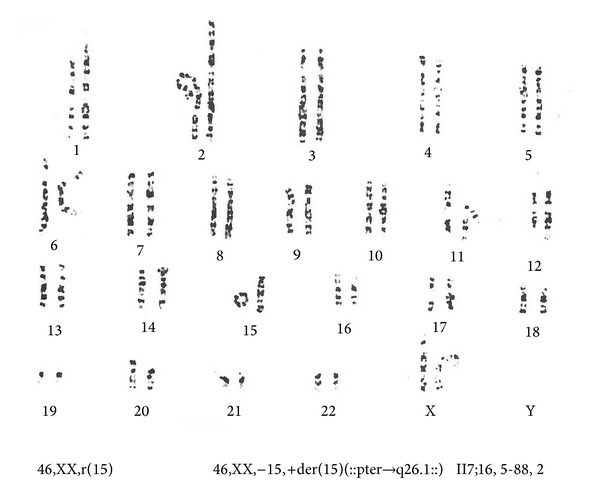
The karyotype of 46,XX, r(15) pattern.

Cytogenetic analyses from 72 hr lymphocyte cultures showed pathological karyotype: mos 45,XX-15[4%]/46,XX,-15,+der(15)(::pter->q26.1::)[96%]. Parental karyotype is normal and according to the parents, the karyotype anomaly was classified as* de novo* (Figure 1). The analysis of a three-generation pedigree has shown no genetic abnormalities or pregnancy losses.

**Figure 2:**
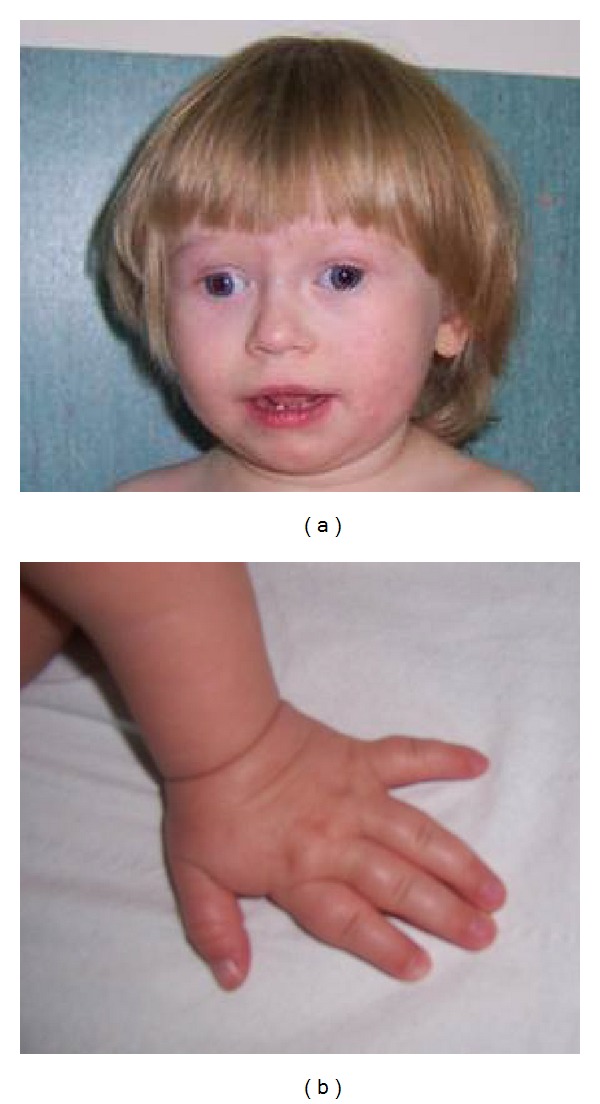
(a) Dysmorphic features of the patient, big exotropia. (b) Fifth finger clinodactyly.

During the first clinical examination at the age of 15 months, extreme short stature was noticed—the girl was 66 cm (−5.6 SD); her weight was also very low: 7 kg (<3 percentile) and her BMI was 15.9 kg/m^2^. A detailed examination revealed several dysmorphic features: hypertelorism, high-broad nasal bridge, short hands and feet, and divergent strabismus (Figures 2(a) and 2(b)). Detailed neuropediatric consultation revealed psychomotor development delay, hypotonia, and a speech development delay; hyperactivity was not noticed. Liver and renal function and anatomy were normal. The imaging of the central nervous system yields normal results. Bone age—assessed according to the Greulich-Pyle method—was 12 months delayed.

In hormonal tests no abnormalities were detected. Thyroid status as well as adrenal hormones was normal. Growth hormone stimulation tests showed normal results (maximal GH peak in L-DOPA test was 11.8 ng/mL; maximal peak in Clonidine test was 15.3 ng/mL). IGF-1 (198 ng/mL at the age 2 years and 11 months) and IGFBP-3 (5.2 ug/mL) [normal range 0.9–4.3 ug/mL] levels were normal. Because of extreme short stature rhGH therapy was introduced at the age of 3 years with the initial dose of 0.035 mg/kg/d. The result of the 1st year of therapy was an increase in growth velocity by 6 cm. Screening for inborn metabolism disorders with the use of GC-MS method showed no abnormalities.

At initial visit (at 15 months of age) the patient presented with signs of amblyopia in the right eye by fixation pattern, hypermetropia of +3.50 diopters (D) in both eyes, and intermittent right eye exotropia of 60 prism diopters (PD) at distance and near in the primary position as well as convergence insufficiency. A dilated fundus exam showed no abnormalities.

**Figure 3:**
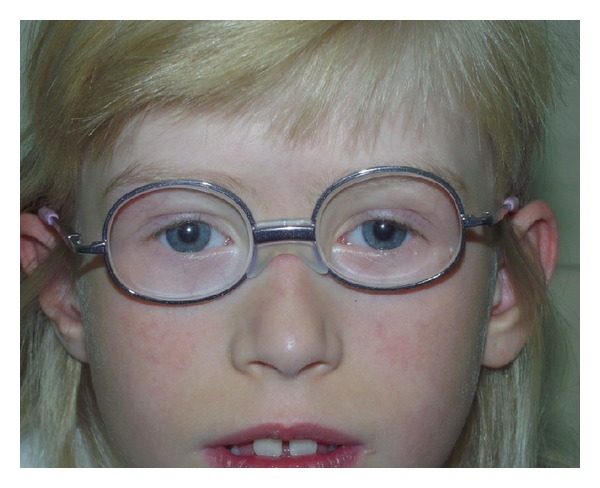
Facial features at age 8 years. Result of ophthalmological treatment: parallel position of the eyeball with final correction OD 5PD, OS 5PD.

Initial management included spectacle correction +1,0 D and 25 PD each eye, nonsquinting eye occlusion (conventional occlusion) for 2 hours per day, and simultaneous undercovering, as well as daily convergence exercises. The lack of parental consent for strabismus operation left conservative treatment as the only option, which proved to be a challenge. At the age of 4 years the best corrected visual acuity (BCVA) was 0.22 OD and 0.33 OS. Remarkable progress in the treatment was seen in the eighth year of therapy governed by the patient's own efforts. The prisms enabled permanent symmetrical stimulation of the retina which leads to the development of normal single binocular vision (Maddox test, filter test, and synoptofor tests). In this case study, the final correction OD 5PD, OS 5PD led to achievement of normal single binocular vision and improvement of visual acuity (1.0 OD/OS) (Figure 3).

## 3. Discussion 

The etiology and pathogenic pathways for developing r(15) are not completely understood. All ring chromosomes are formed by a loss of the terminal fragment of the chromosome and a break-point junction to the terminal region of the short arm of the same chromosome. In this scenario, the size of lost fragment and haploinsufficiency of the missed genes determine the clinical features. Because nearly all of the patients present with loss of different span of the terminal chromosome fragments, determining common genotype-phenotype correlation is impossible. They present with unspecific features, classified by Fryns et al. [10] and Kosztolanyi et al. [11, 12] as the so called “ring phenotype.” Our patient showed in first years of life the clinical features resembling Silver-Russel phenotype (growth deficit, microcephaly, triangular face with typical dysmorphic signs). It is probably due to a loss of one copy of the IGFR-1 gene (Insuline-Like Growth Factor Recepror 1). IGF- like receptor mutations are discussed as one of the main factors influencing the pathogenesis of Silver—Russell Syndrome, mainly characterized by growth deficit [13].

Ring chromosomes in general but particularly r(15) may be unstable structures and in some cases may be lost or duplicated in some cells during embryonic differentiations resulting in new mosaic cell lines; such mechanism could be present in our patient as we observed a monosomy 15 cell line without r(15) in small number of cells [14]. The risk of having next child with pathology increases with mother's age (>age 35) and continues to increase with each year of life. Parental karyotype was normal, and no genetic abnormalities found in siblings consolidate us in a single occurrence of this type of chromosomal disorder in this family.

According to the original report of Jacobsen [1], congenital malformations in ring chromosome 15 patients included eye anomalies (e.g., macular defects, hyperopia, strabismus and heterochromia), ear abnormalities (e.g., dysplastic ears and hearing loss), café-au-lait macules, and cardiac anomalies [2, 6, 7]. Of the genes mapped to distal 15q (http://www.ncbi.nlm.nih.gov/mapview), none have been directly implicated in the etiology of human strabismus. Literature search showed no data in regard to ophthalmologic treatment in children diagnosed with r(15). As far as we know, abovementioned case of achieving normal single binocular vision and improving visual acuity has been presented for the first time.

Delayed psychomotor development and difficult contact with the child certainly had an impact on the length of ocular treatment. As previously mentioned, in this r(15) case study the ophthalmological findings included exotropia and deep amblyopia. Spherical correction of hyperopia and prismatic lenses were utilized to correct the strabismus angle in order to achieve symmetrical stimulation of both retinas at distance and near. At the beginning of therapy, the first given spectacles were purposely of less prismatic value then the angle of squint. Knowing that if we wanted to completely correct the deviation of the eye we would have to prescribe full prism correction, and they would worsen the already poor visual acuity. Therapy has prolonged to several years due to general complications of the congenital defects (typical for ring15) and ocular disorders (strabismus instability, abnormal fixation pattern, deep amblyopia, and convergence insufficiency). An optimistic attitude of both the ophthalmologists and the parents, as well as the cooperation of many specialists in treating such a difficult ring chromosome 15 patient resulted in an optimal positive ocular result.

## Abbreviations

BCVA: Best corrected visual acuity
BMI: Body mass index
D: Diopters
GH: Growth hormone
IGF-1: Insulin-like growth factor 1
IGFBP-3: Insulin-like growth factor binding protein 3
L-DOPA: L-3,4-Dihydroxyphenyalalanine
OD: Right eye
OS: Left eye
r(15): Ring chromosome 15
rhGH: Recombinant human growth hormone
PD: Prism diopters.
